# The Success Story of Gold-Based Catalysts for Gas- and Liquid-Phase Reactions: A Brief Perspective and Beyond

**DOI:** 10.3389/fchem.2019.00691

**Published:** 2019-10-24

**Authors:** Cameron A. H. Price, Laura Pastor-Pérez, Svetlana Ivanova, Tomas R. Reina, Jian Liu

**Affiliations:** ^1^Department of Chemical and Process Engineering Department, University of Surrey, Guildford, United Kingdom; ^2^State Key Laboratory of Catalysis, Dalian Institute of Chemical Physics, Chinese Academy of Sciences, Dalian, China; ^3^Departamento de Química Inorgánica, Universidad de Sevilla, Instituto de Ciencias de Materiales de Sevilla Centro Mixto (US-CSIC), Seville, Spain

**Keywords:** gold, catalyst and catalysis, heterogeneous, gas phase, liquid phase

## Abstract

Gold has long held the fascination of mankind. For millennia it has found use in art, cosmetic metallurgy and architecture; this element is seen as the ultimate statement of prosperity and beauty. This myriad of uses is made possible by the characteristic inertness of bulk gold; allowing it to appear long lasting and above the tarnishing experienced by other metals, in part providing its status as the most noble metal.

Gold has long held the fascination of mankind. For millennia, it has found use in art, cosmetics, metallurgy, and architecture; this element is seen as the ultimate statement of prosperity and beauty. This multiplicity of uses is made possible by the characteristic inertness of bulk gold, which allows it to appear long-lasting and immune to the tarnishing experienced by other metals, in part leading to its status as the most noble of metals. Though its inertness makes bulk gold catalysis impossible, this property disappears when gold is broken down to the nanoscale, in which form it has been found to be incredibly reactive. Although the dependence of the catalytic ability of a material on size is well-known, such an extraordinary increase in activity due to particle size is highly impressive and intriguing, and several different explanations have been provided to explain this characteristic of gold. The explanations are myriad and range from quantum size effects (Valden et al., 1998a,b), to charge transfer with the support (Sanchez et al., 1999; Ricci et al., 2006), to oxygen spill-over (Hammer, 2006; Liu et al., 2006), and even to the oxidation state of Au (González-Arellano et al., 2006; Hutchings et al., 2006). However, it is largely believed that a combination of these factors is responsible for the impressive performance of gold, with particle size being at the root (Hvolbæk et al., 2007).

This trend in size and activity for gold particles was discovered in seminal works by Hutchings (1985) and Haruta et al. (1987) on small, well-dispersed gold nanoparticles, which were found to be highly effective catalysts for both the CO oxidation reaction and the hydrochlorination of acetylene. The high activity in the former reaction is especially surprising, since gold displays endothermic chemisorption energies for oxygen according to a DFT study on first principles, implying an inability toward binding oxygen (Hvolbæk et al., 2007).

Despite this apparent aversion for oxygen, through the exploitation of small (<5 nm) nanoparticles, gold has found itself applied toward oxidation reactions for quite some time, with significant year-on-year increases in the number of related publications (Figure 1). Gold catalysts have found use in various reactions, from CO, hydrocarbon, alcohol, and volatile organic compound (VOCs) oxidation to water gas shift (WGS), hydrogenation of unsaturated compounds and nitroarenes, and hydrochlorination reactions. Furthermore, gold is not confined to an academic setting, finding applications in industrial catalysis as well: the Au-doped Pd catalyst used in the production of vinyl acetate was designed over 50 years ago and is still the industrial standard (Gao and Goodman, 2012), and a AuNiO_x_ core-shell catalyst is known to be used in the production of methyl methacrylate (Suzuki et al., 2013). In fact, it has been determined that the two most limiting factors for industrial gold catalyst usage are (1) catalytic durability under industrial conditions and (2) finding practical methods of synthesis and are not, in fact, purely the cost of gold (Corti et al., 2005). The development process of new catalysts is faced by two serious hurdles: firstly, the new material must offer an advance over existing processes, perhaps using cheaper raw materials (as with vinyl acetate synthesis, where its use replaced acetylene with ethylene), as well as offering breakthrough economics (Teles, 2008; Ciriminna et al., 2016). A recent example of this ideology can be seen in the 2015 announcement by Johnson Matthey that they will begin using their new gold catalyst in the production of vinyl chloride monomer (VCM), which goes on to become PVC. This catalyst will replace the existing HgCl_2_ material used in current VCM/PVC plants, the use of which currently accounts for 50% of the world's mercury usage (Ciriminna et al., 2016).

**Figure 1:**
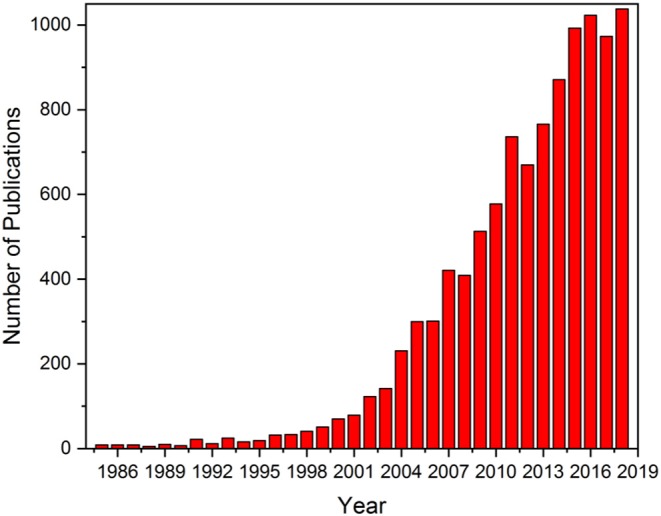
Publication metrics for the period 1985–2018 concerning “Gold catalysts” and “oxidation reactions” as guide strings. Source: Web of Science 29/05/2019.

This special issue aims to discuss some recent advances in the growing number of reactions where gold has found meaningful and developed application over existing materials (Figure 2). Although the applications that fall within the purview of this special issue are many and wide-ranging, herein, we will discuss the recent advances made within these processes. In addition, this perspective also provides some hints on reduction reactions to showcase the versatility of gold catalysis for both oxidative and reductive processes.

**Figure 2:**
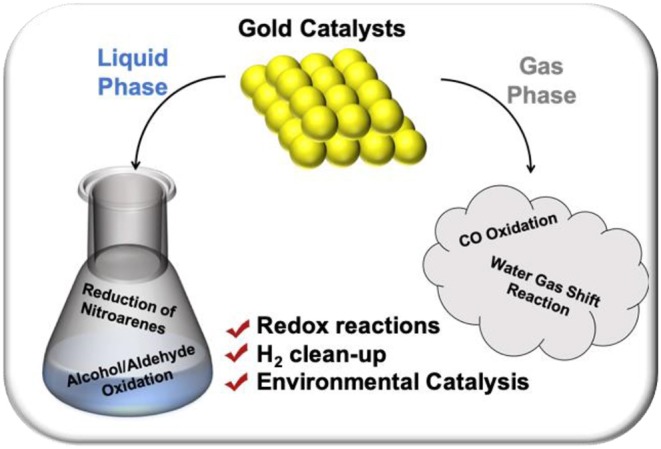
Graphical synopsis of this work.

## Gas-Phase Applications: Examples Based on CO Oxidation and WGS

The use of nanogold within gas-phase oxidation reactions is not novel. In fact, some of the initial work concerning the use of nanogold was reported by Haruta et al. (1987), when they reported its use on a number of transition metal oxides to catalyze both H_2_ and CO oxidation. Over the last decade, this has evolved into the use of nanogold supported on materials with high reducibility (e.g., TiO_2_ and CeO_2_) or doped (e.g., Cu, C, Pd) with numerous other materials to produce top-tier catalysts.

Gold has been found to be more successful in small-scale applications than are conventional materials. The work by Haruta et al. not only found nanogold to be highly effective but demonstrated that the performance of the catalyst was actually increased by the presence of moisture, which became evident in the work of Andreeva et al., which pioneered a highly effective use of an Au/Fe_2_O_3_ catalyst for low-temperature WGS reaction (Andreeva et al., 1996). These works, and those like them at the time, produced the foundation upon which current research on nanogold catalysts for gas-phase reactions is based.

A popular method of developing catalytic materials is the investigation of novel support media, as there is a link between catalytic performance and active phase-support interaction. This is no different for gold catalysts; a 2010 study by Widmann et al. investigated four supports for gold catalysts in the CO oxidation reaction. The study supported the findings of Schubert et al. (2001) in that the interaction with the support and its reducibility played a crucial role in the activity of the catalysts, with “active” or reducible supports leading to more active gold catalysts and “inert” or non-reducible supports producing inactive materials. The activity of the examined materials was concluded to be, in descending order, TiO_2_ > ZrO_2_ > ZnO > Al_2_O_3_. This continues to add credence to the hypothesis that reducible supports offer a crucial synergy with nanogold and participate in the catalytic process, most likely through oxygen adsorption and activation at oxygen vacancies on the support material that are either close to the Au particles or form at interface sites (Widmann et al., 2010).

This latter finding perhaps contributed to a theory of an activity hierarchy of gold species, as supported catalysts often display a variety of structures, such as dispersed atoms, clusters, and nanoparticles. If this is the case, the reported activity of gold catalysts should be relative to the presence of the different species of gold within the sample. This was confirmed in a joint study by Hutchings and Haruta that compared the different activities of a gold catalyst produced in two different ways. Furthermore, when considering the mechanism of CO oxidation, this hierarchy of activity explains why small gold clusters and nanoparticles are active for CO oxidation while bulk or larger particles are not (Hutchings, 2018).

There has been a considerable amount of work done concerning numerous support materials for gold catalysts toward CO oxidation applications, namely, CeO_2_, Fe_2_O_3_, NiO, and Co_3_O_4_, with further studies toting the benefits of mixed metal oxide supports over mono-metal oxides, i.e., CeO_2_-Fe_2_O_3_, TiO_2_-ZrO_2_, and V_2_O_5_-TiO_2_. In addition to CO oxidation reactions, these catalysts have also shown promising results toward the oxidation of volatile organic compounds and the WGS reaction, which are explored in more detail elsewhere (Barakat et al., 2013). The enhanced activity when using these mixed metal oxides could be the result of modified acid-base and redox properties that occur with the incorporation of other metal oxides. Furthermore, this mixing of metallic oxides could also afford altered surface electronic properties to positively affect the absorption and reactivity of reactant molecules (Idakiev et al., 2003).

Originally, the WGS reaction found its industrial use in the production of ammonia (Haber-Bosh process) following its first documentation in 1780. Recently, however, there has been renewed interest in the WGS reaction, which is itself a form of CO oxidation. This attention stems from its potential for producing hydrogen while reducing CO levels in reformate streams, mainly in proton exchange membrane fuel cells (PEMFCs). The standard materials used for this reaction are either iron-chromium oxides, for high-temperature usage, or copper-based, for low-temperature application. The problem with existing Cu catalysts is that they are only suitable for relatively small space velocities, making them unsuitable for portable applications. Meanwhile, iron catalysts suffer from agglomeration at high temperatures and the development of metallic iron from the “active” magnetite phase (Bouarab et al., 2014). Consequently, precious metal catalysts represent significant improvements over the transition metal systems, especially as they demonstrate superior conversion at higher space velocities.

When considering gold catalysts for this process, they are often divided into two sections depending on their support media: reducible and non-reducible.

Conventionally, it is accepted that the use of a reducible support is required to activate water molecules, which is usually considered the rate-determining step for gold catalysis (Carter and Hutchings, 2018). The most recent studies concerning this type of support make use of CeO_2_ (Abdel-Mageed et al., 2017), CeZrO_4_ (Carter et al., 2017; Stere et al., 2017), CeO_2_-Al_2_O_3_ (Reina et al., 2013, 2015), transition metal-doped CeO_2_ (Tabakova et al., 2013), and Cu-ZnO-Al_2_O_3_ (Santos et al., 2017, 2018). Morphology is also beginning to be considered, with a recent work concerning the application of Au@TiO_2_ yolk-shell catalysts toward oxidation reactions. This material was reported to demonstrate high CO oxidation at cryogenic temperatures, which was noted to be part of a trend of increasing activity with decreasing temperature (Zaera, 2018).

A noteworthy study describes the use of non-thermal plasma to activate the water molecule in the gas phase over an Au/CeZrO_4_ catalyst. This adaptation demonstrated high activity at low temperatures, which was attributed to the decoupling of the thermodynamics of the WGS reaction from its kinetics. The latter was achieved by applying a dielectric-barrier discharge activation to the catalyst material, allowing the reaction to proceed at lower temperatures (Stere et al., 2017).

Non-reducible supports, like Al_2_O_3_, SiO_2_, or C, contrary to reducible supports, are unable to activate water molecules or participate in the reaction at all. This makes them generally less active than catalysts containing CeO_2_ or Fe_2_O_3_ (Sandoval et al., 2007; Gil et al., 2011; Shekhar et al., 2012). Catalysts of this design therefore allow for more innovative solutions to enhance the performance of these materials. For example, the use of Na or K ions as promoters catalyzed the water-gas shift at low temperatures to the same (or similar) intrinsic activities as other nano-gold catalysts, regardless of support reducibility (Yang et al., 2014). Similarly, Mo_2_C has been demonstrated to be a highly effective support for low-temperature water-gas shift with gold catalysts, with 4- to 8-fold increases of activity over the conventional Cu/ZnO/Al_2_O_3_ catalyst being reported (Patt et al., 2000; Liu and Rodriguez, 2006; Ma et al., 2017). A notable study in this area concerns the use of Au/MoC catalysts that reported very high levels of activity for low-temperature WGS reaction at 473 K (3.19 mol_CO_${\text{mol}}_{\text{Au}}^{-1}$s^−1^) (Yao et al., 2017).

## Liquid-Phase Applications: Some Insights into Oxidative and Reductive Processes

In addition to these gaseous processes, the application of gold catalysts toward liquid-phase reactions, for instance the oxidation of alcohols and aldehydes, reduction of nitro compounds, and the application of gold nanoparticles toward biological applications, are also well-known. This topic has received significant contributions from Prati et al. concerning the production of Au/C using metallic sols and protecting agents like Polyvinyl pyrrolidone (PVP) and polyvinyl alcohol (PVA) to maintain the nanoscale of the gold particles (Prati and Rossi, 1997, 1998; Prati and Martra, 1999; Porta et al., 2000) for the oxidation of alcohols (diols). These works noted not only the dominating success of the Au/C catalyst over Pd or Pt equivalents in terms of activity and poisoning resistance but also the notable effectiveness for the production of nanogold particles that using metallic sols and protecting agents affords. Numerous additional works concerning nanogold and alcohol oxidation reactions and the reaction pathway have been contributed by Friend et al. (Cremer et al., 2014; Personick et al., 2015, 2017; Siler et al., 2016; Wang et al., 2016; Xu et al., 2018). These provide significant insights into the activity of gold for oxidation reactions [i.e., np(Ag)Au catalysts for the partial oxidation of methanol] (Wang et al., 2016).

As the techniques for the oxidation of alcohol shifted from the use of permanganates and chromates due to environmental concerns, the field began looking for more atom-efficient forms of oxidation reactions that made use of molecular oxygen rather than activated oxygen. The oxidation of alcohols is very challenging under these criteria. However, nanogold was demonstrated to be highly effective for this conversion, with initial works describing the oxidation of 1,2-propanediol into lactic acid via a selective primary alcohol partial oxidation under basic conditions (Porta et al., 2000). This was developed based on a study from Christensen et al. (2006) that has furthered research in this field by using supported gold nanocrystals and milder basic conditions. Commonly reported gold catalysts for this application have been supported on numerous different materials. A recent review (Hui et al., 2019) extensively investigates this topic, highlighting the commonality of support media: C, CeO_2_, TiO_2_, SiO_2_, and Al_2_O_3_, as well as more complex LDH, MOF, and MgCuCr_2_O_4_ materials. Of these, the CeO_2_- and MgCuCr_2_O_4_-supported catalysts were described to be the most effective materials investigated, leading to promising results for alcohol oxidation/reduction processes, owing perhaps to the reducibility or oxygen storage capacity of these materials.

Further to alcohol conversion, gold catalysts are showing promise for converting aldehydes to carboxylic acids using similar gold catalyst systems. Some recent work does detail the effective application of Au catalysts toward oxidizing 2-hexenal (Alshammari, 2016). The study detailed several different support media, for instance, CeO_2_, TiO_2_, and Al_2_O_3_, while also introducing some less reported supports: MnO_2_, SiC, and MgO. The study found these materials to be highly effective for this reaction.

Another reaction that gold catalysts have been found to have significant application toward is the reduction of nitroarenes. This reaction is the most commonly applied form of environmental remediation that removes nitro-compounds, while also being key in the production of amino aromatics. Gold nanoparticles are considered economically viable for this reaction, as the catalytic activity is controllable through the size of the nanoparticles so that high activity can be achieved under relatively mild conditions. A great number of works have discussed the applicability of gold toward these reactions over a variety of novel support media (Corma and Serna, 2006; Corma et al., 2006, 2007a,b, 2009; González-Arellano et al., 2008; Serna and Corma, 2015). In fact, Corma et al. have extensively studied the application of gold catalysts toward targeted hydrogenation, finding them not only superior to existing catalysts in the reduction of other functional groups (González-Arellano et al., 2005; Comas-Vives et al., 2006) but also capable of targeted reduction of amine groups in nitroarenes, even in a heavily substituted molecule (Corma and Serna, 2006; Corma et al., 2007a,b).

When consulting the literature (Qin et al., 2019), the same support materials consistently appear. TiO_2_, SiO_2_, MgO, and Al_2_O_3_ are mentioned, with a number of very recent works focusing on porous carbons and carbon allotropes (Guo et al., 2016; Fu et al., 2017, 2018). Interestingly, however, varied morphologies are being reported (Lee et al., 2008; Huang et al., 2009; Zheng et al., 2013; Chen et al., 2014); yolk-shell variants of Au/SiO_2_ have been reported for the reduction of nitro-phenol, displaying the high performance expected of Au catalysts, detailing a significant increase in TOF (6.6–36 s^−1^) with a reduction in Au core size (104–43 nm) (Lee et al., 2008). Furthermore, these materials displayed a 40% increase in TOF values compared to bare gold nanoparticles of the same size under the same conditions.

One of the greatest draws of gold is the mystery that it still preserves. No matter which reaction (oxidation or reduction), gold is full of potential, willing to serve and still presenting challenges after 30 years of intensive use. For example, (i) the particular relationship of gold with reductive supports and the limited selection of support materials that are synergistic, (ii) its genuine size/structure/activity-dependence, making the understanding of its behavior hard to predict, and (iii) its behavior change in the presence of other metals or under different reaction conditions.

What is important for the near future? It is necessary to:

- Explore the new horizons opened by the intensive introduction of gold metal into biorefinery reactions.
- Explore its full potential in the reverse WGS reaction, decoupling the thermodynamics and kinetics and using the excellent synergy that exists between copper and gold.
- Find a support material able to stabilize nanogold in the liquid-phase reactions where its true potential still remains under-explored.
- Spark new ideas and assist in their economical implementation in industrial processes.

It is easy to fall in love with this metal, and it is easy to spend a lifetime trying to understand how a shiny piece of metal could be the best electrocatalyst for oxygen reduction, the best heterogeneous catalyst for CO oxidation, and the best homogeneous catalyst for the production of some key added-value chemicals.

Allow gold to enter your life, and you will never regret it.

## Author Contributions

CP produced and wrote the article. LP-P edited the article. SI edited the article. TR and JL conceptualist, edited the article, and supervision.

### Conflict of Interest

The authors declare that the research was conducted in the absence of any commercial or financial relationships that could be construed as a potential conflict of interest.
